# Mechanisms of Competitive Adsorption Organic Pollutants on Hexylene-Bridged Polysilsesquioxane

**DOI:** 10.3390/ma8095275

**Published:** 2015-08-31

**Authors:** De-Rong Lin, Li-Jiang Hu, Bao-Shan Xing, Hong You, Douglas A. Loy

**Affiliations:** 1 College of Food Science, Sichuan Agricultural University, Ya’an 625014, China; 2 State Key Laboratory of Urban Water Resource and Environment, Harbin Institute of Technology, Harbin 150090, China; E-Mail: yuhu0104@hit.edu.cn; 3 Department of Plant, Soil & Insect Sciences, University of Massachusetts Amherst, MA 01003, USA; E-Mail: bx@umass.edu; 4 Departments of Materials Science & Engineering and Chemistry and Biochemistry, University of Arizona, Tucson, AZ 85721, USA

**Keywords:** hexylene bridged polysilsesquioxane, organic pollutants, pore blockage, competition adsorption sites, competitive adsorption

## Abstract

Hexylene-bridged periodic mesoporous polysilsesquioxanes (HBPMS) are a promising new class of adsorbent for the removal of organic contaminants from aqueous solutions. These hybrid organic-inorganic materials have a larger BET surface area of 897 m^2^·g^−1^ accessible through a cubic, isotropic network of 3.82-nm diameter pores. The hexylene bridging group provides enhanced adsorption of organic molecules while the bridged polysilsesquioxane structure permits sufficient silanols that are hydrophilic to be retained. In this study, adsorption of phenanthrene (PHEN), 2,4-Dichlorophenol (DCP), and nitrobenzene (NBZ) with HBPMS materials was studied to ascertain the relative contributions to adsorption performance from (1) direct competition for sites and (2) pore blockage. A conceptual model was proposed to further explain the phenomena. This study suggests a promising application of cubic mesoporous BPS in wastewater treatment.

## 1. Introduction

Mesoporous organosilicas, such as Hexylene-bridged periodic mesoporous polysilsesquioxanes (HBPMS), have good potential for effectively removing a wide range of organic pollutants from water. Organic pollutants include organic compounds varying in molecular weight between a few hundred Daltons (peptides) and one hundred thousand Daltons (proteins) [1] with a wide range of functionality and shapes. However, the efficacy of adsorption decreases substantially with increasing concentration for all sorbents. Presently, there are two mechanisms for inhibition of pollutant adsorption: direct site-competing (SC) and pore blockage (PB). Direct competition for available adsorption sites, and the resulting reduction of adsorption capacity, is mainly caused by organic pollutants with lower molecular weights [2]. In contrast, pore blockage is mainly caused by larger molecules, which adsorb in larger pores and constrict, possibly completely blocking the entrance to smaller pores. 

Despite previous research efforts [3,4,5], the actual adsorption process is extremely complex and is not fully understood. This is particularly the case for adsorbents being applied to polluted water samples, due to their complexity. One traditional method to characterize adsorption is to use model compounds instead of actual polluted water samples [6]. Yang *et al.* [7] used 2,4-dichlorophenol (DCP), naphthalene (NAP), and 4-chloroaniline(PCAN) as simple models for lower molecular weight aromatic pollutants (<200 g/mol) in water that contribute to direct competition. In an affecting atrazine (215.7 g/mol) adsorption competition study, compounds with molecular weights between 200 to 700 g/mol were found to be mostly responsible for pore constriction adsorption [4]. Similarly, Newcombe *et al.* [8,9] evaluated the competitive effect of organic pollutant fractions and found that their smallest fraction, with molecular weight (MWw) of 949 g/mol, respectively, was the most detrimental to 2-methylisoborneol (168 g/mol) adsorption capacity and removal rates. 

The objective of this study was to examine the adsorption of phenanthrene (PHEN) onto high surface area, cubic HBPMS, and the competitive adsorption effects of DCP and nitrobenzene (NBZ). We also discussed if the direct SC and PB influences the amount of organic compounds in HBPMS. 

## 2. Experimental and Methods

### 2.1. Materials

Synthesis of 1,6-bis(triethoxysilyl)hexane (BESH) was carried out according to literature procedure [10]. PHEN was obtained from Syngenta Crop Protection, Inc. (Greensboro, NC, USA) and American Radiolabeled Chemicals, Inc. (St. Louis, MO, USA). DCP (>99.5%) was purchased from Shanghai Reagent Co. (Shanghai, China). NBZ (>99%) was purchased from Sigma-Aldrich Chemical Co. (St. Louis, MO, USA). 

### 2.2. Synthesis of Hexylene BPS

A modified version of the synthesis described by Lin *et al.*, was followed [11,12,13]. A mixture of N-(3-trimethyl-ammoniumpropyl)hexadecylammonium dibromide (4.6 mmol, 2.44 g) and NaOH (18.3 mmol, 0.732 g) in 55 g warm deionized water was stirred to form a clear solution for 0.5 h at 40 °C. Then, BESH (8 mmol, 3.28 g) was added dropwise and the stirring continued for 24 h at ambient temperature to generate a homogeneous solution. Heating this solution at 95 °C for 6–7 h formed a white precipitate. The suspension was aged in a high-density polyethylene bottle at 80 °C for 96 h without stirring. A white powder was recovered by suction filtration without water washing and dried at ambient temperature (1.968 g). Surfactant was removed from the synthesized mesoporous organosilica material by stirring in a solution of 150 mL ethanol and 5 mL 37% hydrochloride acid for 6 h at 50–60 °C. The extracted precipitate was separated by suction filtration and dried in air (1.148 g grams, yield = 61%).

### 2.3. Characterization of Hexylene BPS

**Figure 1:**
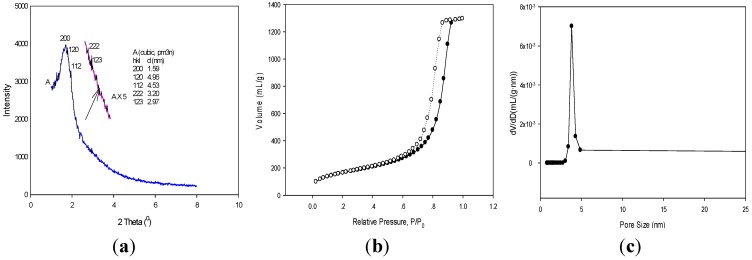
Small-angle X-ray diffraction (XRD) (**a**) nitrogen adsorption-desorption, (**b**) and pore size distribution (**c**) of the cubic *Pm3n* Hexylene-bridged periodic mesoporous polysilsesquioxanes (HBPMS). hkl: miller indices.

Powder X-ray diffraction patterns were recorded on a Philips X’Pert instrument (Philips Panalytical XRD, Almelo, The Netherlands) using monochromatic Cu Kα radiation (λ = 1.5418 Å). The unit cell parameter for the *Pm3n* symmetry group was evaluated using the interplanar spacing *d* of the (110) XRD peak: *a*_0_ = $\sqrt{2}$ *d*_110_. Nitrogen adsorption-desorption isotherm was measured on a Quantachrome Instrument Corporation Autosorb-1 analyzer (Quantachrome Instrument Corporation, Boynton Beach, FL, USA). The samples were degassed at 200 °C using vacuum below 20 mmHg. The Brunauer-Emmet-Teller (BET) specific surface areas were calculated from the adsorption data in the relative pressure range from 0.05 to 0.235 (Figure 1b). The density functional theory (DFT) [14,15] methods were used for calculations of pore diameter and pore volume.

### 2.4. Organic Pollutants

For each of a new batch put into use, PHEN, NBZ, and DCP isotherm in distilled water were performed for quality control. Stock solutions of PHEN were prepared for each batch by dissolving solid PHEN in distilled water to make a concentration around 10–15 mg/L. The stock solution was mixed on a stir plate for a day until all solids had been dissolved. They were stored in a fridge at 4 °C and were later used to prepare all test solutions. 

Aqueous concentration of solutes was determined by mixing 2.5 mL of sample with 18 mL of scintillation cocktail solution (Ecoscint, National Diagnostics, Inc. Atlanta, Georgia, USA) in a 20-mL glass vial and analyzed by a liquid scintillation counter (Tricarb Model 1600A, Packard Instrument Co., Downers Grove, IL, USA). 

### 2.5. Single-Solute Adsorption Isotherms

Adsorption of PHEN, NBZ, and DCP was carried out using the conventional bottle-point technique [16] to which the equilibrium data were correlated to Freundlich (FM), Sips, and Toth models. Single solute adsorption isotherms were performed using the conventional bottle-point technique at room temperature. A test solution was prepared by spiking stock solution of the test compound to background solution and mixing it on a stirplate. The test solution was dispensed into white isotherm bottles, each of which had pre-weighed HBPMS inside. Bottles were then sealed with screw caps and Teflon tape and put in a shaker for seven days. Preliminary tests had proved a duration of seven days is enough to reach equilibrium. PHEN samples were taken by drawing 5 mL of solution from a bottle using a gas-tight syringe and pushing it through a 13 mm 0.45 μm nylon filter. The first 2.5 mL was discarded and the next exact 2.5 mL was collected in a 20-mL vials. 

### 2.6. Competitive Isotherms

Competitive isotherm tests were performed in a similar way as single solute isotherms, except that the test solutions were made by adding PHEN stock solution into filtered, distilled water. The different initial concentrations of PHEN were realized by varying the volume of the stock solution added. Equilibrium samples at seven days for PHEN and for DCP and NBZ were taken and analyzed. The 30-day isotherms (data not shown) were almost identical to the seven-day isotherms for HBPMS, showing that a contact time of seven days is enough for PHEN, DCP, and NBZ adsorption to reach equilibrium on the HBPMS. The sampling process for PHEN can be found in the previous paragraph and the PHEN samples were taken by filtering 60 mL of solution through 0.45 μm nylon filters with 40 mL being collected. Please also refer to the previous sections for analysis processes for individual compounds. For single-solute PHEN isotherms in distilled water, an initial concentration of around 100 mg/L was used for HBPMS. Such high initial concentrations were used to assure detectable equilibrium concentrations after adsorption. Lower initial concentrations were used for PHEN adsorption in distilled water because DCP and NBZ competition greatly reduced adsorption capacity for PHEN. For HBPMS, two initial concentrations of PHEN, 1 and 10 mg/L, were used in the presence of DCP and NBZ, which produced different levels of competition and provided higher reliability in later model fitting. 

Three different models were used in this study. They are as follows:

FM model (Equation (1)):

(1) $$Q_{\text{e}}=K_{\text{f}}C_{\text{e}}^{n}$$

where *Q*_e_ and *C*_e_ again refer to amount adsorbed/unit mass of adsorbent and equilibrium concentration, respectively. *K*_f_ ((mg/g)/(mg/L)*^n^*) is the Freundlich affinity coefficient, and *n* is the Freundlich exponential coefficient.

Sips isotherm model (Equation (2)) [17]:

(2) $$Q_{\text{e}}=\frac{Q_{\text{m}}bC_{\text{e}}^{1/n}}{1+bC_{\text{e}}^{1/n}}$$

where *Q*_e_ (mg/g) is the equilibrium sorbent concentration, *Q*_m_ parameter is relevant with adsorption capacity, *a*_s_ (*b*) constant related to energy of adsorption and 1/*n* is exponent.

Toth isotherm model (Equation (3)) [18]:

(3) $$Q_{\text{e}}=\frac{Q_{\text{m}}C_{\text{e}}}{{\left(K_{\text{T}}+C_{\text{e}}^{n}\right)}^{1/n}}$$

where *Q*_e_ (mg/g) is the equilibrium sorbent concentration; *K*_T_ is the Toth model constant, and *n* the Toth model exponent (0 < *n* ≤ 1).

All experiments were performed in triplicate. All model parameters with their standard errors were determined by a commercial software program (SPSS 18.0, SPSS Inc, Chicago, IL, USA). Mean-weighted-square-errors (*MWSE*), and correlation coefficients (*r*^2^) were used to evaluate the goodness of fit.

## 3. Results and Discussion

### 3.1. Preparation and Characterization of HBPMS

The preparation of the cubic phase HBPMS has been reported elsewhere. The material was confirmed to be cubic *Pm3n* symmetry by small-angle X-ray diffraction (XRD) and transmission electron microscopy (TEM). In the small-angle XRD there are peaks at (200), (210), (211), (222) and (123) that correspond to d-spacing of 1.59, 4.96, 4.53, 3.20 and 2.97 nm and a lattice parameter of 11.68 nm (Figure 1a). This is consistent with a cubic *Pm3n* structure, similar to Santa Barbara amorphous material (SBA-1) reported earlier by Kim *et al.* and Huo *et al.* [19,20]. 

The N_2_ adsorption/desorption isotherm of the HBPMS sample is consistent with a high surface area and mesoporous material. DFT analysis of pore size distribution reveals a very narrow distribution of pores at 3.82 nm. The BET surface area, pore size, and pore volume of the sample are 897 m^2^/g, 3.82 nm, 0.96 cm^3^/g (Figure 1b,c), respectively. Such mesoporous architecture with a large surface area and pore volume plays an important role in catalyst design for improving the adsorption of reactant molecules [21,22,23,24]. 

### 3.2. Adsorption Isotherms of HBPMS

**Figure 2:**
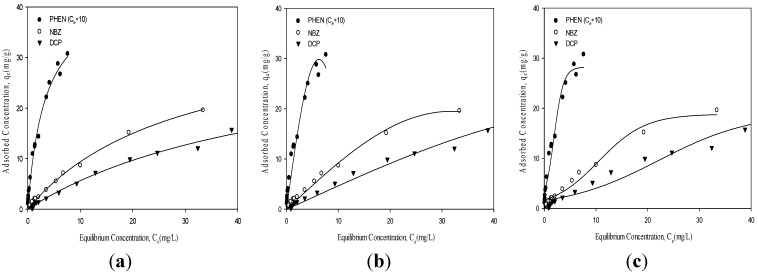
Fit to adsorption data of phenanthrene (PHEN), nitrobenzene (NBZ), and 2,4-Dichlorophenol (DCP) by the HBPMS using the (**a**) Freundlich (FM), (**b**) Sips and (**c**) Toth models.

Figure 2 is a graph of the single solute isotherms for PHEN, DCP, and NBZ adsorbing onto a 100 mg sample of HBPMS. To get the points, small doses of a solute are introduced to the sorbent and the amount of adsorbed solute (*y*-axis) and the amount of solute left in the solution are determined by measuring the later by scintillation. The FM model had a better fit than the Sips and Toth models as indicated by the fitting-adjusted square of the correlation coefficient (*r*_adj_^2^) (Table 1). Since the FM model has been recognized as one of the most powerful available models for dealing with aqueous adsorption on energetically heterogeneous surfaces, the *r*_adj_^2^ (>0.9870) of the internal validation suggest the good fit of the FM model. Therefore, we use FM model parameters to correlate adsorption behavior with the properties of adsorbates and adsorbents. 

The adsorption isotherms of these organic pollutants were also simulated with the FM isotherm model (Equation (1) and line drawn through the points). At low equilibrium concentration, there are many active sites and the adsorption isotherm shows a steep rise as most of the material is adsorbed. Once the active sites are covered (pores start to fill), the slope of isotherm decreases dramatically. The curve for PHEN is significantly to the left of the isotherms for DCP and NBZ, indicating that the HBPMS has a greater affinity for the former. A comparison of the adsorption of NBZ and that of DCP shows that more NBZ than DCP is adsorbed on HBPMS even when the initial concentration of DCP is higher, indicating that NBZ has a much larger intrinsic affinity for the HBPMS surface than DCP does. The non-linearity of the isotherms indicates that the adsorbent is heterogeneous with adsorption sites of different activities [4]. 

**Table 1:** Comparison of two-parameter and three-parameter isotherms for different organic pollutants. PHEN: phenanthrene; NBZ: nitrobenzene; DCP: 2,4-Dichlorophenol.

Isotherms	PHEN	NBZ	DCP
Freundlich			
*K*_f_	29.10 ± 0.93	1.41 ± 0.04	0.88 ± 0.02
*n*	0.58 ± 0.02	0.88 ± 0.03	0.89 ± 0.03
*r*_adj_^2^	0.9871	0.9916	0.9886
Sips			
*Q*_m_	35.85 ± 1.27	24.81 ± 1.11	23.63 ± 0.87
*a*_s_	0.063 ± 0.002	0.049 ± 0.001	0.033 ± 0.001
*1*/*n*	0.533 ± 0.02	0.556 ± 0.02	0.834 ± 0.03
*r*_adj_^2^	0.9574	0.9881	0.9844
Toth			
*Q*_m_	69.39 ± 3.01	49.33 ± 2.17	46.98 ± 1.72
*K*_T_	1.67 ± 0.09	1.19 ± 0.03	3.86 ± 0.07
*n*	0.248 ± 0.01	0.256 ± 0.01	0.729 ± 0.03
*r*_adj_^2^	0.9639	0.9704	0.9500

This HBPMS has a three-dimensional (3D) porous structure with hydrophilic channels whose surfaces are decorated by uncoordinated O atoms in the ligands and coordinated water molecules. Despite the comparable sizes of PHEN, NBZ, and DCP molecules, only DCP molecules can enter the channels. This was attributed to the fact that DCP molecules can form strong H-bonds with the O donors on the pore surface, whereas PHEN and NBZ cannot. This material has a stable interpenetrated 3D framework structure with one-dimensional (1D) channels. Even though the pore size is large, PHEN cannot diffuse into the mesopores, whereas DCP and NBZ molecules can. This unusual adsorption selectivity was accounted for by the strong interactions of the pore blockage, which blocks other molecules from passing into the pore. 

### 3.3. Competitive Adsorption between PHEN and Neutral DCP/NBZ

**Figure 3:**
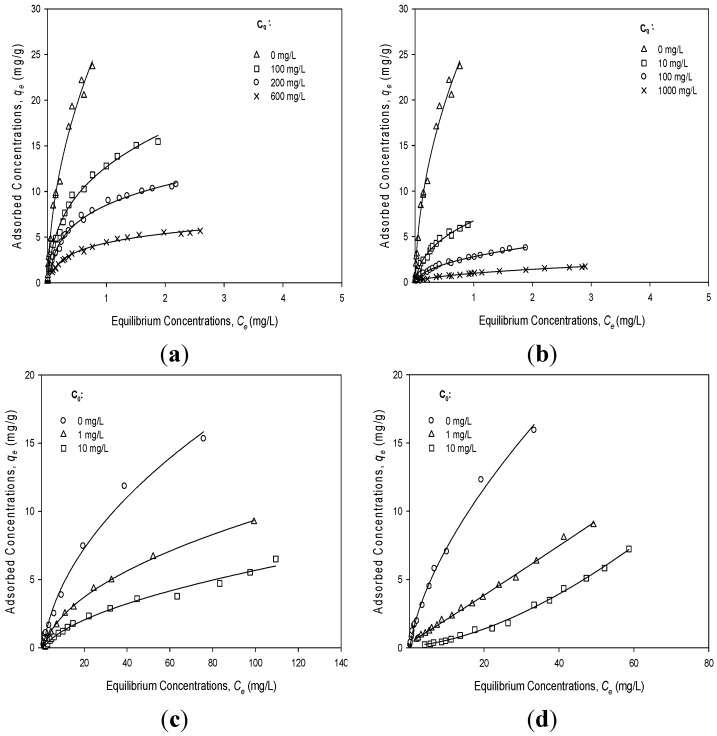
Single-solute and bi-solute isotherms of PHEN and DCP and NBZ on HBPMS and the fitting of the FM model: (**a**) PHEN isotherms with or without NBZ (at pH 7.0) as the competitor. (**b**) PHEN isotherms with or without DCP (at pH 7.0) as the competitor. (**c**) DCP isotherms with or without PHEN (at pH 7.0) as the competitor. (**d**) NBZ isotherms with or without PHEN (at pH 7.0) as the competitor. *C*_0_ (mg/L) is the added dose of the competitors.

Competitive isotherms (Figure 3a–d) are generated with mixtures of two solutes being adsorbed onto the HBPMS. As in the single solute isotherms, the subject solute is added in small doses to 100 mg of the HBPMS and allowed to equilibrate before measuring the concentration of solutes before and after adsorption and solutes in solution. In these experiments, the amount of subject solute (PHEN in Figure 3a,b, DCP in Figure 3c, and NBZ in Figure 3d) is kept at a relatively low dose level. The competition is from an excess of a second solute that is added to the solution at the beginning. If the subject solute is more strongly adsorbed than the competing solute, its curve should be relatively unaffected by the distortion. If the competing solute binds competitively, then the subject isotherm will be shifted more strongly to the right and may be attenuated on the initial segment of the isotherm with the steepest slope. In each graph, the first (upper left curve in each case) curve is the single solute isotherm for the solute of interest. The additional curves are the isotherms for the solute of interest in the presence of relatively high concentrations (100, 200 and 600 ppm) of competing solute. In Figure 3a,b, the solute of interest is PHEN and the competing solutes are NBZ and DCP, respectively. In comparing the two, it is clear that DCP has a significantly greater impact on the adsorption isotherms of PHEN than NBZ. In Figure 3c, the solute of interest is the DCP with PHEN as the competitor. In Figure 3d, the solute of interest is NBZ and PHEN is the competitor solute. 

**Figure 4:**
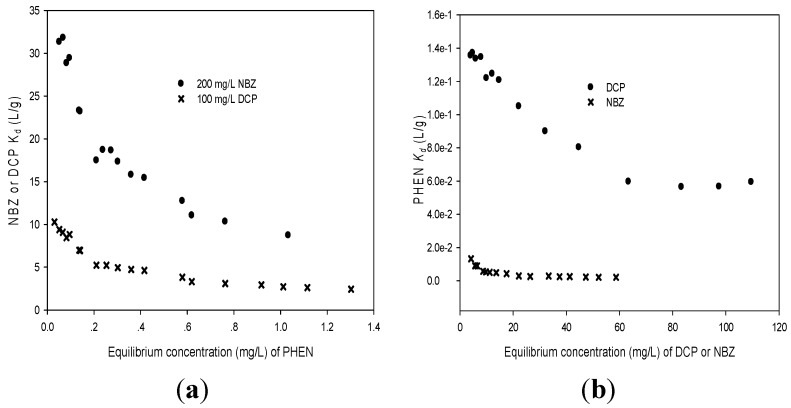
Competition between PHEN and neutral DCP and NBZ on HBPMS: (**a**) *K*_d_ of 200 mg/L NBZ or 100 mg/L DCP as a function of equilibrium PHEN concentration; (**b**) *K*_d_ of 10 mg/L PHEN as a function of equilibrium DCP or NBZ concentration.

Adsorption coefficients (*K*_d_ = *q*_e_/*C*_e_) of NBZ and DCP at an initial concentration of 200 mg/L, 100 mg/L, respectively, decrease significantly with PHEN as the competitor (Figure 4a). Adsorption coefficients (*K*_d_) of PHEN at an initial concentration of 10 mg/L also decrease significantly with DCP as the competitor, but decrease slowly with NBZ as the competitor (Figure 4b). The FM model was used to predict the competitive adsorption isotherm of NBZ and DCP when PHEN is present (see Figure 3). Figure 3 and Table 2 show PHEN isotherms with neutral DCP/NBZ in background solutions and DCP/NBZ isotherms with PHEN, respectively. The decrease of *K*_f_ and *n* values of PHEN isotherms could be attributed to the pore blockage effect of HBPMS on PHEN adsorption capacity. The adsorption capacity for PHEN was determined from the equilibrium concentrations of PHEN. The presence of DCP/NBZ slowly reduced the PHEN adsorption capacity of HBPMS. Since NBZ, DCP and PHEN could adsorb mainly in mesopores, the large mesopore surface area of HBPMS did have some noticeable effect on the competition.

**Table 2:** Freundlich (FM) model for PHEN and DCP and NBZ in non-competitive adsorption and in competitive adsorption on Hexylene-bridged periodic mesoporous polysilsesquioxanes (HBPMS).

Primary-Sorbate (Competitor)	*C*_0_^a^ (mg/L)	*K*_f_^b^	*n* ^c^	*MWSE* ^d^	*r*_adj_^2^
DCP	0	0.88 ± 0.02	0.67 ± 0.02	0.0504	0.9886
DCP (PHEN)	1	0.81 ± 0.04	0.63 ± 0.02	0.0373	0.9900
DCP (PHEN)	10	0.79 ± 0.05	0.58 ± 0.03	0.0265	0.9910
NBZ	0	1.41 ± 0.04	0.70 ± 0.02	0.0410	0.9916
NBZ (PHEN)	1	1.32 ± 0.03	0.68 ± 0.05	0.0315	0.9932
NBZ (PHEN)	10	1.28 ± 0.05	0.65 ± 0.07	0.0298	0.9977
PHEN	0	29.10 ± 0.93	0.58 ± 0.02	0.0890	0.9870
PHEN (NBZ)	100	27.69 ± 1.13	0.55 ± 0.04	0.0203	0.9897
PHEN (NBZ)	200	25.97 ± 1.44	0.50 ± 0.07	0.0197	0.9911
PHEN (NBZ)	600	23.85 ± 1.61	0.42 ± 0.05	0.0128	0.9970
PHEN (DCP)	10	28.09 ± 1.24	0.53 ± 0.04	0.0323	0.9901
PHEN (DCP)	100	27.73 ± 1.76	0.49 ± 0.03	0.0231	0.9963
PHEN (DCP)	1000	24.96 ± 2.01	0.41 ± 0.06	0.0196	0.9979

^a^ *C*_0_ is the initial concentration of competitors. ^b^ *K*_f_ ((mg/g)/(mg/L)*^n^*). ^c^ *n* is the Freundlich exponential coefficient. ^d^ *MWSE* is mean-weighted-square-errors, equal to (1/ν) × ((*q*_measured_ − *q*_modeled_)^2^/*q*_measured_^2^), where ν is the amount of freedom; ν = *N* − 3 for FM model, *N* is the number of data points.

PHEN isotherms with neutral DCP/NBZ adsorption capacity were lower than that obtained only by PHEN, probably because some DCP/NBZ adsorbed in pores that are too small for PHEN to access. It can be derived from Figure 4a that a lower initial concentration of PHEN resulted in lower adsorption coefficients (*K*_d_ = *q*_e_/*C*_e_) in NBZ than those in DCP. This is consistent with the observation that the strength of competition is dependent on the relative concentration of the two competing components [25]. 

An important mechanism was identified in recent research by Yang *et al.* [7] who showed that adsorption of site coverage compounds caused an increase in the adsorbed DCP during adsorption. This increase was attributed to the reduced sorption sites on the pore surface as more and more of DCP’s preferred surface sites were occupied by HBPMS and, thus, PHEN, NBZ, and DCP could take place with less interaction with the pore surface. These authors developed a conceptual model that incorporated both the increase in adsorption capacity caused by the site coverage of HBPMS and the decrease in adsorption capacity caused by the pore blocking effect of HBPMS.

Many studies have been conducted to investigate the mechanisms involved in the adsorption of organic pollutants on mesoporous materials, with a special focus on the roles of pore blocking [5], site coverage [7], and hydrophobic interactions [26]. The pore blocking and site coverage between the hydroxyl functional groups of DCP and the HBPMS surfaces were thought to be the dominant interaction mechanism. Other studies showed that the hydrophobic effects followed by the π-bonding interaction with HBPMS could be the dominant mechanism controlling the chemical adsorption of organic pollutants onto HBPMS surfaces.

## 4. Conclusions

Cubic HBPMS materials that have more surface area in this pore size range had a smaller PB effect on organic pollutant adsorption. The FM equation that describes the enhanced surface adsorption coefficient for organic pollutants as a function of the loading of the site-covering compounds was found to be independent of either the mesoporous HBPMS material type or the competing compound type. The adsorption capacity of the HBPMS materials are relative to the pore size, competitive adsorption sites, and pore blocking. The FM model is applicable for HBPMS’ competitive adsorption of organic pollutants. The pore blocking, competition sites, hydrophobic interactions, and π–π bonding have very important roles in the adsorption of organic pollutants. They are being applied to pact beds/columns that are very different from the mesoporous HBPMS application regarding flow rate, contact time, and mixing conditions. Small-scale column tests may be able to predict the PHEN, NBZ and DCP breakthrough in the presence of HBPMS; it can be seen as the best bench-scale method to study the simultaneous adsorption of heterogeneous contaminant systems. On the other hand, HBPMS materials are pressure-resistant, so they may be used in small-scale column tests with extremely high backpressures.
